# 

**Published:** 1896-06

**Authors:** 


### EDITORIAL.

### Change of Place.

The annual meeting of the Southern Dental Association will be held at Asheville, N. C., on the 28th of July, 1896, the headquarters being at Battery Park Hotel. The intention some time ago was that this meeting should be held at Lookout Mountain, but learning that ample accommodations could not there be obtained, a change of place was found to be necessary. The indications are that the attendance will be very large. It is, however, a little unfortunate, perhaps, that this meeting occurs but a week before the annual meeting of the American Dental Association at Saratoga, N. Y., and also the meeting of three other bodies at the same time and place, at each of which many of the members of the Southern Association ought to be present, and in which many of them are greatly interested. It may be, however, that the plan is, on the part of many, to go direct from Asheville to Saratoga. We hope this may be the case. The time for holding the Southern Association meetings will, undoubtedly, prevent many of the profession fron the more northern portions of our country from attending the southern meetings, which is a matter of regret, and we would suggest that all of the southern brethren go to Saratoga, and thus overcome this unfortunate circumstance as far as possible.

### Inter-State Dental Meeting.

The States of Iowa, Nebraska, Missouri and Kansas will hold an Inter-State Dental Meeting at Excelsior Springs, Missouri, June 23 to 26, 1896, inclusive. The Executive Committee having the matter in charge have made extensive preparations and very complete arrangements for this joint meeting. The accommodations at Excelsior Springs are unsurpassed in the West, and, without doubt, this will be a very enjoyable occasion. Preparation has been made for an extensive clinic, of which Dr. H. J. McKellops, of St. Louis, is supervisor-general, and every one who knows McKellops, will readily understand the character of the clinics that will be given. Clinicians will please bear in mind that they should come prepared with instruments for any work they propose doing. Engines and lathes will be provided by the supervisor. The display of electrical appliances will be a special feature. Inter-State meetings constitute a new feature in dental society work, and in every instance, so far, where attempted, have proven to be eminently successful. Elements enter into this class of meetings which are not found in the small

State dental meetings, and certainly not in those of a more local character. We shall expect to hear of grand results from this meeting.

### Notice.

The thirty-eighth annual meeting of the Indiana State Dental Association will be held at Indianapolis, Ind., in the new Indiana Dental College building, commencing Tuesday, June 30th, 1896, at 10:00 A. M. All members of the profession are invited to attend.

The State Board of Dental Examiners will meet at the same time and place.

M. A. Mason, D.D.S., *Secretary*,
Fort Wayne, Indiana.

